# L‐serine: a neglected amino acid with a potential therapeutic role in diabetes

**DOI:** 10.1111/apm.12987

**Published:** 2019-09-02

**Authors:** Laurits J. Holm, Karsten Buschard

**Affiliations:** ^1^ The Bartholin Institute Department of Pathology Rigshospitalet Copenhagen N Denmark

**Keywords:** Deoxysphingolipids, diabetes‐related complications, L‐serine, type 1 diabetes, type 2 diabetes

## Abstract

L‐serine is classified as a non‐essential amino acid; however, L‐serine is indispensable having a central role in a broad range of cellular processes. Growing evidence suggests a role for L‐serine in the development of diabetes mellitus and its related complications, with L‐serine being positively correlated to insulin secretion and sensitivity. L‐serine metabolism is altered in type 1, type 2, and gestational diabetes, and L‐serine supplementations improve glucose homeostasis and mitochondrial function, and reduce neuronal death. Additionally, L‐serine lowers the incidence of autoimmune diabetes in NOD mice. Dietary supplementations of L‐serine are generally regarded as safe (GRAS) by the FDA. Therefore, we believe that L‐serine should be considered as an emerging therapeutic option in diabetes, although work remains in order to fully understand the role of L‐serine in diabetes.

Diabetes mellitus is a group of metabolic disorders characterized by hyperglycaemia. This is caused by a lack of insulin in type 1 diabetes or a combination of insulin resistance and reduced insulin production in type 2 diabetes and gestational diabetes 1. The number of diabetes patients has according to The World Health Organization (WHO) almost quadrupled since 1980 to an estimated 422 million in 2014 2. The rise in type 2 and gestational diabetes can to a large degree be explained by increasing obesity rates while it remains elusive why type 1 diabetes incidence is rising 3. All forms of diabetes have tremendous effects on whole‐body metabolism, and tight control of blood glucose is needed to reduce the risk of diabetes‐related complications, for example, peripheral neuropathy including retinopathy, nephropathy, and heart attacks. However, even with tight control of blood glucose, most patients will develop complications, with diabetic neuropathy, 90% of all diabetes patients, being the most common 4. Thus, there is a high need for therapeutic approaches to reduce the incidence of diabetes and its related complications.

Amino acids have received attention in relation to diabetes with especially branched‐chain and aromatic amino acids being reported to affect type 2 diabetes risk 5. In this review, we will focus on the otherwise neglected amino acid L‐serine. L‐serine is a polar amino acid which was first discovered in 1865 by Cramer as a component of raw silk 6. Since then, L‐serine has been described as a vital amino acid in all living organism. L‐serine was originally classified as a non‐essential amino acid; however, it was quickly discovered that vertebrates under certain circumstances cannot synthesize enough to meet cellular demands 7, 8. A more appropriate term might, therefore, be ‘conditional non‐essential amino acid’. Dietary intake of L‐serine is consequently necessary even in healthy individuals. Our dietary intake of L‐serine is distributed among many types of food with the highest contributions coming from L‐serine rich foods such as eggs, soy, cheese, and nuts.

L‐serine can be derived from four sources: (i) dietary intake, (ii) biosynthesis from glycolytic intermediate 3‐phosphoglycerate, (iii) glycine, and (iv) turnover of protein and proteins and phospholipids (Fig. 1) 9. The relative contribution of these sources is unknown and varies depending on tissue type and developmental stage 7. The liver, for instance, gets most of its L‐serine from glycine while the kidneys synthesize it from 3‐phosphoglycerate 10, 11.

**Figure 1:**
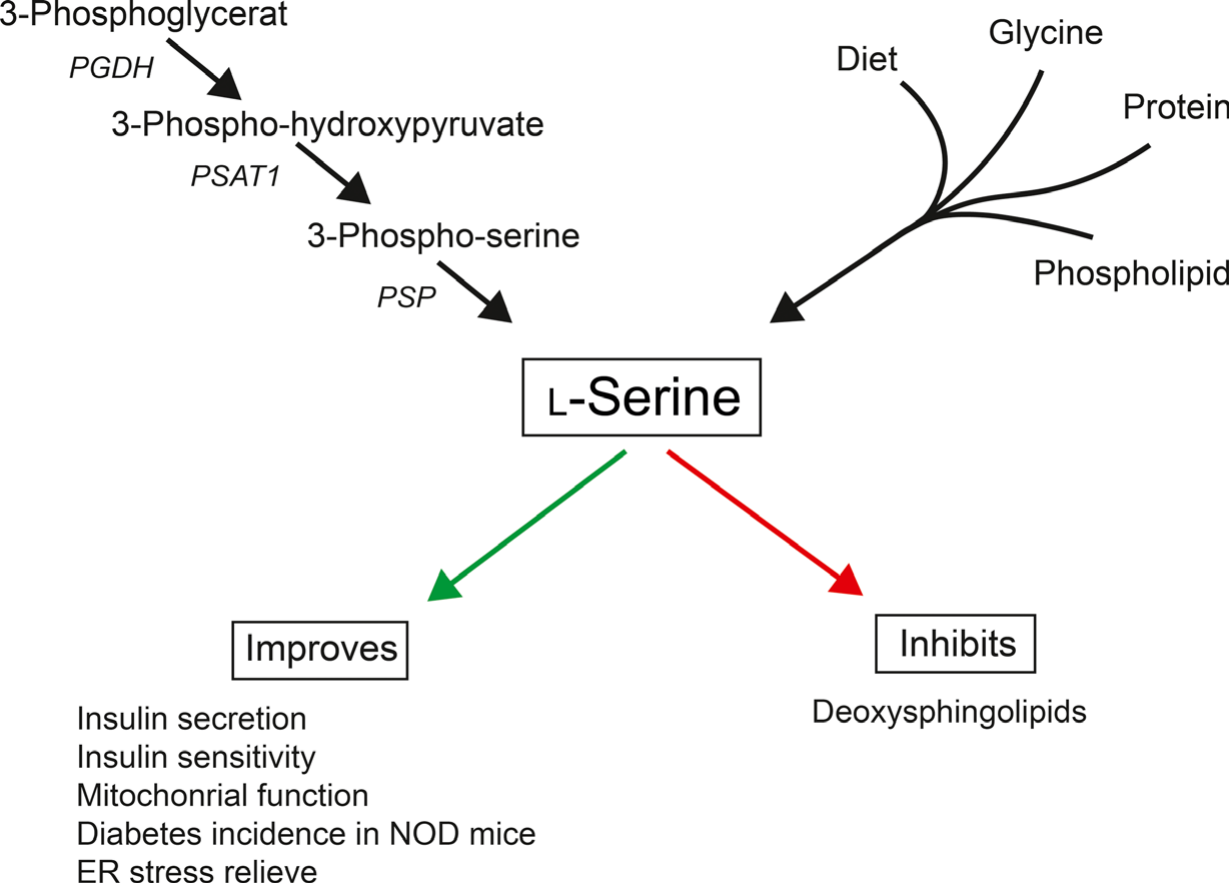
L‐serine metabolism and activities. The pathways for the creation of L‐serine are shown with *de novo* synthesis by *phosphoglycerate dehydrogenase* (PGDH), *phosphoserine aminotransferase* (PSAT), and *phosphoserine phosphatase* (PSP). Other sources of L‐serine are through diet or from glycine or by the turnover of proteins and phospholipids. L‐serine has various biological effects, mentioned here are the ones with relation to diabetes. NOD, non‐obese diabetic.

Biosynthesis of L‐serine is catalysed by the enzymes phosphoglycerate dehydrogenase (*PGDH*), phosphoserine aminotransferase (*PSAT*), and phosphoserine phosphatase (*PSP*). Flux is mainly regulated by *PSP,* which is regarded as the rate‐limiting step in serine biosynthesis 12. Regulation of synthesis is controlled by cellular demands with upregulation of the enzymes in rapidly proliferating tissue and in cell cultures of L‐serine starved cells 13, 14. A protein‐restricted diet has in rats been shown to double L‐serine concentration in blood, due to increased *de novo* synthesis 15. Two enzymes in L‐serine biosynthesis have been linked to insulin sensitivity. *PSAT* expression and liver L‐serine levels are reduced in leptin receptor‐deficient (db/db) mice and high‐fat diet (HFD)‐induced diabetic mice 16. Insulin signalling and sensitivity were subsequently shown to be improved following overexpression of *PSAT*. Inhibition of *PSP* was similarly shown to cause inappropriate serine dephosphorylation of substrates resulting in insulin resistance in rat adipocytes 17.

Defects in L‐serine biosynthesis or transport have profound developmental consequences. Neu–Laxova syndrome is a rare disease caused by a mutation in the *PGDH* gene, if homozygous it causes prenatal or early postnatal death limp malformation and neural tube defects 18. Other defects in L‐serine biosynthesis can cause other developmental and intellectual disabilities 8, 9. Treatment with L‐serine in these diseases improves some symptoms however not completely, suggesting that L‐serine deficiency can give lasting neurological damage 8, 19.

L‐serine is known to participate in various metabolic pathways besides being a constituent of proteins. For instance, D‐serine, an enantiomer of l‐serine, made by serine racemase is a ligand for the N‐methyl‐D‐aspartate (*NMDA*) receptor and mediates neuronal excitation 20. It should be noted that supplementation of D‐serine reduces insulin secretion in mice and that serine racemase is linked to the development of diabetic retinopathy 21, 22. L‐serine itself is a neuronal trophic factor promoting growth, differentiation, and elongation of cultured neurons 9. These beneficial effects might be connected to L‐serine being required for the synthesis of phosphatidylserine and sphingolipids, of which especially sphingolipids are suggested to play a major role in diabetes development 23, 24, 25. Additionally, then L‐serine is involved in the formation of NADPH, nucleotides, and as a carbon source for methylations 9, 15, 26. L‐serine participates in a metabolic network connected to cell proliferation with some cancer cells having increased *PGDH* expression, while L‐serine dietary restrictions have therapeutic effects on tumour growth 15, 26.

A recent study looking at amino acid concentration in 5181 non‐diabetic men aged between 45 and 73 years from the Finnish town Kuopio in relation with the development of type 2 diabetes found that a high L‐serine concentration is correlated to improved insulin secretion and sensitivity 27. L‐Serine is also associated with an improved glucose tolerance after a 2‐h oral glucose tolerance test, although no correlation was found to the development of type 2 diabetes. Another link between L‐serine and blood glucose homeostasis comes from the non‐obese diabetic (NOD) mice, a type 1 diabetes mouse model, in which we found that L‐serine supplementation reduces insulitis and diabetes incidence (43%) compared to controls (71%) 28. This was associated with an improved glucose tolerance, reduced HOMA‐IR, and a small reduction in average glycemic level. Thus, suggesting that L‐serine supplementation could be used to both treat and prevent diabetes. We have identified two L‐serine transporters (*SLC1A4* and *SLC7A10*) with reduced mRNA levels in islet from individuals with newly diagnosed type 1 diabetes, while polymorphisms in the promoter of L‐serine transporter *SLC1A5* (odds ratio 1.16) are genetically linked to the risk of developing of type 1 diabetes 25.

L‐serine concentration has in several studies been found to be significantly altered in diabetes. A study of children with type 1 diabetes and non‐diabetic controls found that the concentration of L‐serine in plasma was decreased by 42% in patients 29. Similar studies of patients with type 2 diabetes have found reduced L‐serine concentration in blood 30, 31. The postprandial L‐serine concentration in response to a standardized meal differs between type 2 diabetes patients and non‐diabetic controls, with patients having a reduced concentration of L‐serine in blood, while controls have an increased concentration 32. Finally, then two studies have reported conflicting results showing a decreased or increased concentration of L‐serine in the blood of individuals with gestational diabetes 33, 34. Reduced L‐serine levels are likely not the result of reduced insulin production as treatment with streptozotocin (a beta‐cell toxin) leads to an increased L‐serine level 22 weeks later in diabetic mice 35.

There is a lack of studies on how a disturbed L‐serine metabolism could affect the development of diabetes and related complications, and so we currently rely on studies of other diseases. An intriguing possibility is that an altered L‐serine metabolism could lead to the production of a novel type of sphingolipids, termed deoxysphingolipids 36. Deoxysphingolipids are known to induce apoptosis in a beta‐cell line and primary islets and to impair neuronal function 36. Deoxysphingolipids are formed during the first step of sphingolipid synthesis when the enzyme serine palmitoyltransferase (*SPT*) instead of L‐serine uses alanine or glycine as alternative amino acid substrates. Patients with primary L‐serine deficiency have increased amounts of deoxysphingolipids, and L‐serine supplementation is known to reduce the concentration of deoxysphingolipids 37, 38. Deoxysphingolipids are enriched in plasma from type 2 diabetes patients while there is no difference between controls and type 1 diabetes patients 39. It is in this regard interesting that the studies describing a reduced L‐serine concentration in type 2 diabetes patients did not report reductions in blood alanine (one study found increased alanine concentration 31) meaning that the alanine/L‐serine ratio in type 2 diabetes patients would favour the formation of deoxysphingolipids 30, 31. Mutations in *SPT* cause hereditary sensory neuropathy type 1 (HSAN1), in which patients have axonal neuropathy with many similarities to diabetic neuropathy 40. Reducing deoxysphingolipids concentration by increasing L‐serine concentration improved neuronal function in a mouse model of HSAN1 and relieved neuropathy in diabetic rats 40.

Another link to a possible role of L‐serine in preserving neurons comes from studies of Alzheimer's disease and amyotrophic lateral sclerosis (ALS), in which clinical trials with L‐serine are ongoing. 8 A phase 1 human trial for ALS showed that patients receiving the highest dose of 30 g/day had a reduced rate of functional loss compared to control patients 41.

L‐serine is believed to protect neurons by upregulating the ER chaperone *PDI* (protein disulphide isomerase), which is involved in refolding misfolded proteins 42. Also, L‐serine seems to induce activation of the unfolded protein response (UPR) as a mechanism to adapt to ER stress by removal of accumulating and aggregating proteins 43. ER stress and the accumulation of misfolded proteins have otherwise been suggested to be involved in the development of type 1 diabetes 44. Another mechanism by which a lack of L‐serine could affect diabetes development is by affecting mitochondrial function, which is known to influence both insulin secretion and sensitivity 45. It was in cancer cells found that a lack of L‐serine leads to increased mitochondrial fragmentation and an altered mitochondrial metabolism with compromised fatty acid oxidation and reduced glucose and glutamine catabolism 26. This was likely the results of L‐serine deprivation causing an altered sphingolipid metabolism.

The US FDA has for human consumption classified L‐serine as generally regarded as safe (GRAS) for use as a food additive, under 8.4% of the total dietary protein (CFR Title 21 Section 17.230.18). As such, we propose that therapeutic options that restore L‐serine concentrations in individuals with diabetes should be considered as a safe way to prevent and treat diabetes and diabetes‐related outcomes.

The work was supported by Kirsten and Freddy Johannsen's fond.

## Conflict of Interest

The authors declare that there is no duality of interest associated with this manuscript.

## Authors Contributions

LJH wrote the manuscript with input from KB. LJH and KB revised the final version. Both authors have read and approved the final manuscript.
